# Increase in oxidative stress levels following welding fume inhalation: a controlled human exposure study

**DOI:** 10.1186/s12989-016-0143-7

**Published:** 2016-06-10

**Authors:** Halshka Graczyk, Nastassja Lewinski, Jiayuan Zhao, Jean-Jacques Sauvain, Guillaume Suarez, Pascal Wild, Brigitta Danuser, Michael Riediker

**Affiliations:** 1 Institute for Work and Health, University of Lausanne and Geneva, Lausanne, CH-1066 Switzerland; 2 Department of Chemical and Life Science Engineering, Virginia Commonwealth University, Richmond, VA 23284 USA; 3 Department of Environmental Health, Harvard T. H. Chan School of Public Health, Harvard University, 665 Huntington Ave., Boston, MA 02115 USA; 4 Department of Scientific Management, National Institute for Research and Security, INRS, Vandoeuvre, 54500 France; 5 SAFENANO, IOM Singapore, Singapore, 048622 Singapore

**Keywords:** Oxidative stress, Welding fume, 8-OHdG, Hydrogen peroxide, Malondialdehyde, Apprentice welders, Tungsten Inert Gas (TIG), Occupational exposure

## Abstract

**Background:**

Tungsten inert gas (TIG) welding represents one of the most widely used metal joining processes in industry. It has been shown to generate a large majority of particles at the nanoscale and to have low mass emission rates when compared to other types of welding. Despite evidence that TIG fume particles may produce reactive oxygen species (ROS), limited data is available for the time course changes of particle-associated oxidative stress in exposed TIG welders.

**Methods:**

Twenty non-smoking male welding apprentices were exposed to TIG welding fumes for 60 min under controlled, well-ventilated settings. Exhaled breathe condensate (EBC), blood and urine were collected before exposure, immediately after exposure, 1 h and 3 h post exposure. Volunteers participated in a control day to account for oxidative stress fluctuations due to circadian rhythm. Biological liquids were assessed for total reducing capacity, hydrogen peroxide (H_2_O_2_), malondialdehyde (MDA), and 8-hydroxy-2′-deoxyguanosine (8-OHdG) concentrations at each time point. A linear mixed model was used to assess within day and between day differences.

**Results:**

Significant increases in the measured biomarkers were found at 3 h post exposure. At 3 h post exposure, we found a 24 % increase in plasma-H_2_O_2_ concentrations ([95%CI: 4 % to 46 %], *p* = 0.01); a 91 % increase in urinary-H_2_O_2_ ([2 % to 258 %], *p* = 0.04); a 14 % increase in plasma-8-OHdG ([0 % to 31 %], *p* = 0.049); and a 45 % increase in urinary-8-OHdG ([3 % to 105 %], *p* = 0.03). Doubling particle number concentration (PNC) exposure was associated with a 22 % increase of plasma-8-OHdG at 3 h post exposure (*p* = 0.01).

**Conclusion:**

A 60-min exposure to TIG welding fume in a controlled, well-ventilated setting induced acute oxidative stress at 3 h post exposure in healthy, non-smoking apprentice welders not chronically exposed to welding fumes. As mass concentration of TIG welding fume particles is very low when compared to other types of welding, it is recommended that additional exposure metrics such as PNC are considered for occupational risk assessments. Our findings highlight the importance of increasing awareness of TIG welding fume toxicity, especially given the realities of welding workplaces that may lack ventilation; and beliefs among interviewed welders that TIG represents a cleaner and safer welding process.

**Electronic supplementary material:**

The online version of this article (doi:10.1186/s12989-016-0143-7) contains supplementary material, which is available to authorized users.

## Background

Occupational scenarios provide a unique setting to assess nanoparticle (NP) exposure and dose response related to the generation of oxidative stress in humans. Welding fume particles, in particular metallic NPs, have gained increased attention due to their potential for triggering oxidative stress reactions, and preliminary evidence that welding fumes may induce free radical activity has been demonstrated [1, 43]. It has been shown that certain metals in welding fumes can produce ROS such as hydroxyl radicals (·OH), superoxide anion (O_2_^•-^), singlet oxygen (^1^O_2_), and H_2_O_2_ [60, 63]. The ROS activity of welding fume particles has also been shown to be size dependent, with particles of the fine and ultrafine range (PM0.1–2.5 and PM0.1) generating higher ROS concentrations when compared with coarse PM10 (PM2.5-10) [9]. In the alveoli, ROS can react quickly with surrounding tissue, damage cell components and launch a cascade of local and systemic responses, which may lead to disease [56].

Of various welding processes, Tungsten Inert Gas (TIG) welding is of particular interest for occupational health due to its propensity to generate metallic welding fume particles almost exclusively at the nanoscale [5, 42]. Due to its exceptionally strong and high quality welds, TIG welding has become one of the most popular welding methods in various industrial sectors [20, 36]. A recent characterization study of TIG welding fume particles found that mean ROS production potential at the breathing zone of welders in controlled, ventilated conditions exceeded average concentrations previously found in traffic polluted air [23]. However, despite the increase in TIG welding and evidence that it may generate acellular ROS, there is limited data available for oxidative stress biomarker concentrations in welders exposed to TIG welding fume.

While the International Agency for Research on Cancer (IARC) categorizes welding fumes in Group 2B (possibly carcinogenic to humans), it has also noted that an unexplained reason for lung cancer risks still exists, which in turn demands more research on the generation of radical ROS and oxidative DNA damage in humans, for each of the numerous welding processes [30, 31]. However, existing studies on oxidative stress in welders face methodological drawbacks which hamper comprehensive assessments of exposure to ROS response. Epidemiological studies in industrial settings often lack complete exposure assessments, or are often unable to control for numerous welding parameters, making it difficult to disambiguate between heterogeneous aerosols. On the other hand, laboratory based studies often involve robotic welders and acknowledge that welding fume characteristics may substantially differ from occupational environments where fumes are generated by human welders [7].

To address this gap, the aim of this study was to assess, under controlled conditions, the effect of TIG welding fume exposure on the time course changes of multiple oxidative stress biomarkers in the EBC, blood, and urine of apprentice welders before and at several time points post-exposure. We hypothesized that inhaled TIG welding fume NPs would initiate oxidative stress in the alveoli, which would be first manifest in the EBC, followed by an increase in oxidative stress markers in the circulation and urine. To test this hypothesis, we assessed the EBC, blood and urine of 20 apprentice welders exposed to TIG welding fume for total reducing capacity, MDA, H_2_O_2,_ and 8-OHdG before and at several time points after exposure.

## Results

A total of 23 volunteers were recruited from local welding apprentice schools, of which 3 were excluded for medical reasons, resulting in 20 volunteers who completed the study. Apprentice welders had on average less than one-year exposure to TIG welding fume, or any other types of welding fume, at the time of participating in the study. Volunteer characteristics are summarized in Table 1.

**Table 1:** Summary of volunteer characteristics (*N* = 20)

Variable	Mean ± SD	Range
Age	17.4 ± 2	15–24
Weight (kg)	76.8 ± 18.7	55–137
Height (cm)	176.7 ± 6.1	162–187
BMI	24.5 ± 5.6	17.6–42.3
FEV1 (L)	4.2 ± 0.5	3.4–5.2
Volume EBC collected (ml)	1.69 ± 0.32	1.01–2.69

EBC values report volume EBC collected in 10 min sampling period

Table 2 presents the welding fume characterization results as measured at the breathing zone (BZ) of the volunteers (*N* = 20) during the 60-min welding task. Full exposure characterization results have been previously summarized in [23]. Briefly, we found that across all volunteers, a majority of the particles (92 %) had measured GMDs below 100 nm, with a mean PNC of 1.69E + 06 particles/cm^3^.

**Table 2:** Mean and range of welding fume characterization parameters as measured during the 60 min welding task at the BZ of the volunteer

Variable	Mean	Range
PNC (particles/cm^3^)	1.69E + 06	8.69E + 05–3.85E + 06
GMD (nm)	45	29–108
Gravimetric mass PM_4_ (mg/m^3^)	0.716	0.25–2.29
ROS production potential (nmol/m^3^)	16.89	0.53–31.2
Elements (mg/m^3^):		
Al	0.386	0.03–1.233
W	0.205	0.012–0.684
Si	0.074	0.007–0.201
Na	0.019	0–0.049
Mg	0.015	0–0.029
Ce	0.007	0.001–0.018
Fe	0.006	0.002–0.015

Elements reported represent the top most abundant elements present on filters collected at the BZ (w/w concentration >1 %)

### Oxidative stress biomarkers

Table 3 presents a summary of oxidative stress biomarker concentrations measured in each biological liquid at each time point on exposure days. A corresponding summary for biomarker concentrations on control days can be found in Additional file 1: Table S3.

**Table 3:** Exposure days: Median concentrations of oxidative stress biomarkers in EBC, plasma and creatinine corrected urine (respective unit/g creatinine), presented by time point

		EBC	PLASMA	CREATININE CORRECTED URINE
	Time	Median (range)	Median (range)	Median (range)
Total Reducing Capacity [a.u.]	T1	26 (15–31.5)	162.2 (120–197.3)^a^	1555.4 (789.2–3311.7)
	T2	27 (13–56.7)	161.8 (107.7–210.3)^a^	2082.1 (1061.2–3388.7)
	T3	26.2 (15.7–34)	167.3 (116.5–245.3)^a^	2118.7 (987.7–4201.8)
	T4	26.6 (15–55)	173.5 (99.5–233.7)^a^	2355.0 (1351.8–3843.8)
H_2_O_2_ [μM]	T1	0.08 (˂LOD- 0.3)	4.6 (1.8–11.7)	12.5 (5.3–209.9)
	T2	0.07 (˂LOD- 0.6)	4.4 (2.1–10.8)	19.9 (5.8–104.3)
	T3	0.05 (˂LOD-0.2)	4.4 (2.0–11.6)	19.5 (5.7–129.9)
	T4	0.07 (˂LOD-0.3)	4.4 (2.0–15.7)	39.9 (6.4–135.8)
MDA [nM]	T1	3.8 (˂LOD-30.0)	103.6 (53.7–212.0)	280.2 (151.5–1012.5)
	T2	6.4 (˂LOD-18.3)	97.7 (64.1–282.2)	288.3 (126.9–634.3)
	T3	5.0 (˂LOD-34.9)	94.5 (71.6–476.6)	272.7 (108.1–513.9)
	T4	3.2 (˂LOD-16.8)	108.0 (63.6–529.6)	236.8 (103.6–415.9)
8-OHdG [μg/l]	T1	–	3.8 (˂LOD -15.2)	1.7 (˂LOD -4.8)
	T2	–	3.9 (˂LOD -26.7)	1.5 (˂LOD -3.1)
	T3	–	4.2 (˂LOD -34.2)	1.8 (˂LOD -19.5)
	T4	–	4.5 (˂LOD -39.2)	2.1 (˂LOD -33.1)

EBC was not assessed for 8-OHdG concentrations. ^a^Total reducing capacity concentration was measured in whole blood and not plasma

The percentage of samples below the limit of detection (LOD) for each biomarker in each biological liquid is presented in Additional file 2: Table S1. It is worth noting that 27.8 % of EBC samples assessed for MDA were below the LOD, and 48.8 % of EBC samples assessed for H_2_O_2_ were below the LOD (see full summary of MDA and H_2_O_2_ concentrations in EBC by study day and timepoint in Additional file 3: Table S2). During the pilot study, it was found that the ELISA assay used was not sensitive enough to detect 8-OHdG concentrations in collected EBC, and 8-OHdG results are presented for plasma and for urine only.

**Fig. 1:**
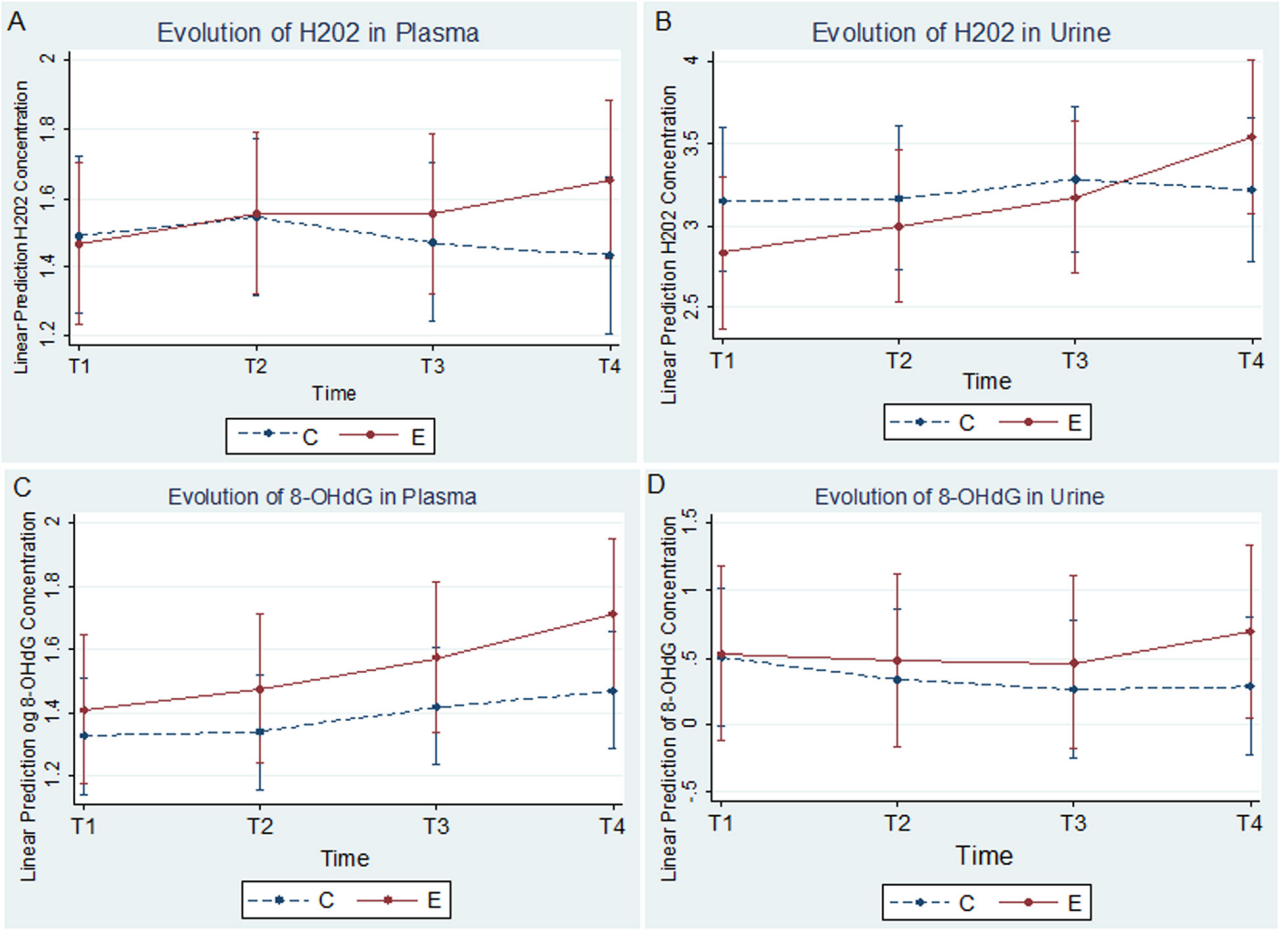
Evolution of H_2_O_2_ and 8-OHdG concentrations in plasma and urine. Evolution of H_2_O_2_ (**a** and **b**) and 8-OHdG (**c** and **d**) concentrations in plasma and urine over time points on both control and exposure days. Solid line represents exposure days while dotted line represents control days

Table 4 presents the results of the linear mixed model used for assessing the time course changes of oxidative stress biomarkers in EBC, plasma and urine, as percentages with 95 % CI and *p*-value. The full summary of these results presented as coefficients with standard error and *p*-value can be found in Additional file 4: Table S4. We found that there was a 24 % significant increase in plasma-H_2_O_2_ concentrations between T1 and T4 ([95 % CI: 4 %; 48 %], *p* = 0.01, Fig. 1a) and a 91 % increase in urinary-H_2_O_2_ concentrations between T1 and T4 ([95 % CI: 2 %; 258 %], *p* = 0.04, Fig. 1b). We also found that there was a 14 % significant increase in plasma-8-OHdG concentrations between T1 and T4 ([95 % CI: 0 %; 31 %], *p* = 0.049, Fig. 1c) and a 45 % increase in urinary-8-OHdG concentrations between T1 and T4 ([95 % CI: 3 %; 105 %], *p* = 0.03, Fig. 1d). There was no significance difference in the time course changes of neither MDA concentrations, nor total reducing capacity concentrations, an indicator of antioxidant action, in any of the biological liquids. Moreover, we did not find a correlation between the time course changes of total reducing capacity and the time course changes of H_2_O_2_, MDA or 8-OHdG.

**Table 4:** Linear mixed model results of the time course changes (i.e., evolution over time) of oxidative stress biomarkers in EBC, plasma and urine, presented as percentages, with 95 % CI and *p*-value

		EBC	PLASMA	CREATININE CORRECTED URINE
	Time	Exponentiated coefficients with 95 % CI (*p*-value)	Exponentiated coefficients with 95 % CI (*p*-value)	Exponentiated coefficients with 95 % CI (*p*-value)
Log-Total Reducing Capacity [a.u]	T1	–	–	–
	T2	−1 % [−18 %;19 %], (0.89)	−5 % [−13 %;4 %], (0.31)^b^	−4 % [−24 %;22 %], (0.75)
	T3	5 % [−13 %;26 %], (0.63)	2 % [−7 %;12 %], (0.63)^b^	12 % [−12 %;42 %], (0.36)
	T4	3 % [11 %;61 %], (0.77)	0.1 %[−9 %;10 %], (0.99)^b^	24 % [−2 %;58 %], (0.08)
Log- H_2_O_2_ [μM]	T1	–	–	–
	T2	12 % [−38 %;103 %], (0.72)	5 % [−12 %;25 %], (0.57)	17 % [−38 %;119 %], (0.62)
	T3	−29 % [−60 %;29 %], (0.26)	14 % [−4 %;36 %], (0.14)	23 % [−34 %;131 %], (0.51)
	T4	−14 % [−52 %;56 %], (0.62)	24 % [4 %;48 %], (0.014)^a^	91 % [−2 %;258 %], (0.043)^a^
Log-MDA [nM]	T1	–	–	–
	T2	−19 % [−58 %;57 %], (0.53)	−4 % [−25 %;23 %], (0.74)	2 % [−25 %;40 %], (0.89)
	T3	−0.8 % [−49 %;92 %], (0.98)	−0.4 % [−22 %;27 %], (0.97)	8 % [−21 %;48 %], (0.63)
	T4	−39 % [−68 %;19 %], (0.15)	−6 % [−26 %;21 %], (0.65)	5 % [−23 %;44 %], (0.76)
Log-8-OHdG [μg/l]	T1	–	–	–
	T2	–	4 % [−9 %;20 %], (0.52)	12 % [−21 %;57 %], (0.54)
	T3	–	6 % [−8 %;21 %], (0.41)	19 % [−16 %;68 %], (0.33)
	T4	–	14 % [0 %;31 %], (0.049)^a^	45 % [3 %;105 %], (0.033)^a^

^a^Indicates significant (*p* < 0.05) increase at the time point as compared to T1 ^b^Total reducing capacity concentration was measured in whole blood and not plasma

### Biomarker concentrations matched with exposure data and welding technique

#### PNC

**Fig. 2:**
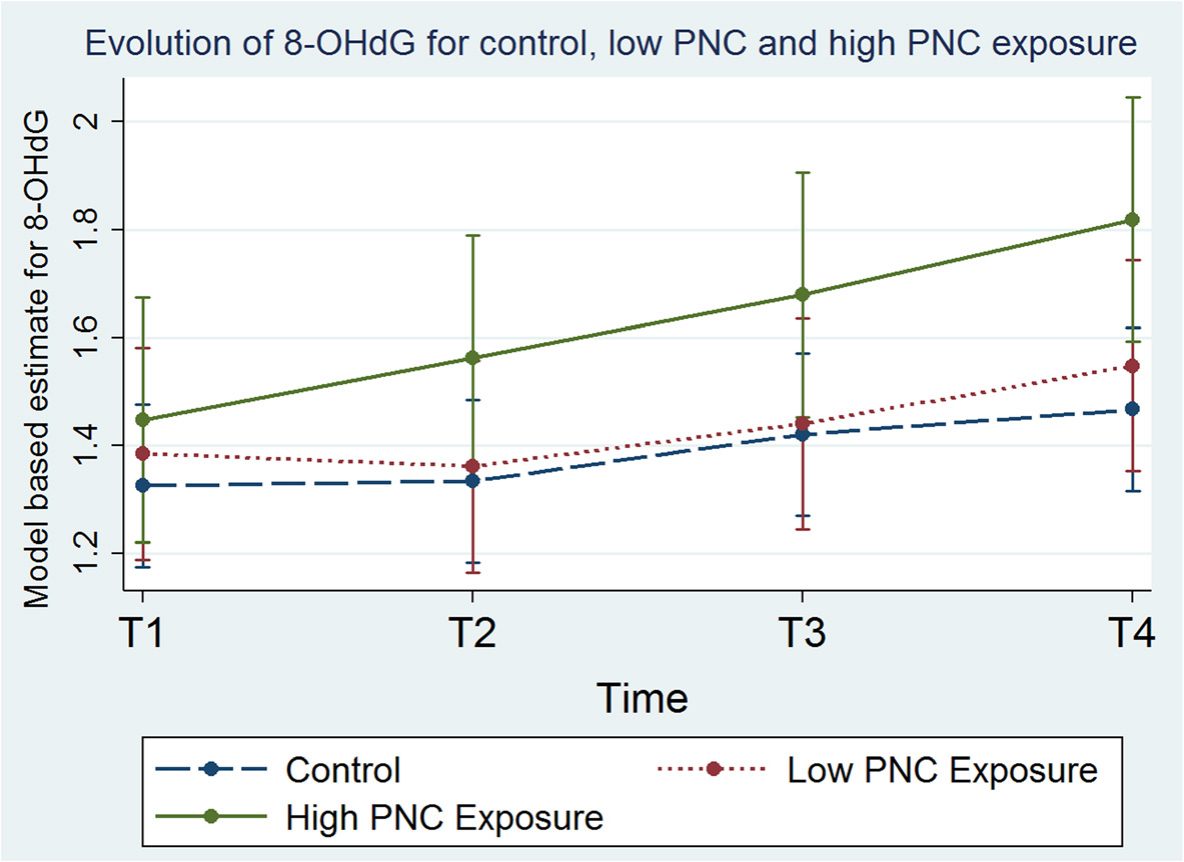
Evolution of 8-OHdG for control, low and high PNC exposure. Evolution of plasma-8-OHdG over time points including data for the control group, low particle number concentration (PNC less than median of 1.6E + 06 particles/cm^3^) group, and high particle number concentration (PNC more than median, 1.6E + 06 particles/cm^3^)

**Fig. 3:**
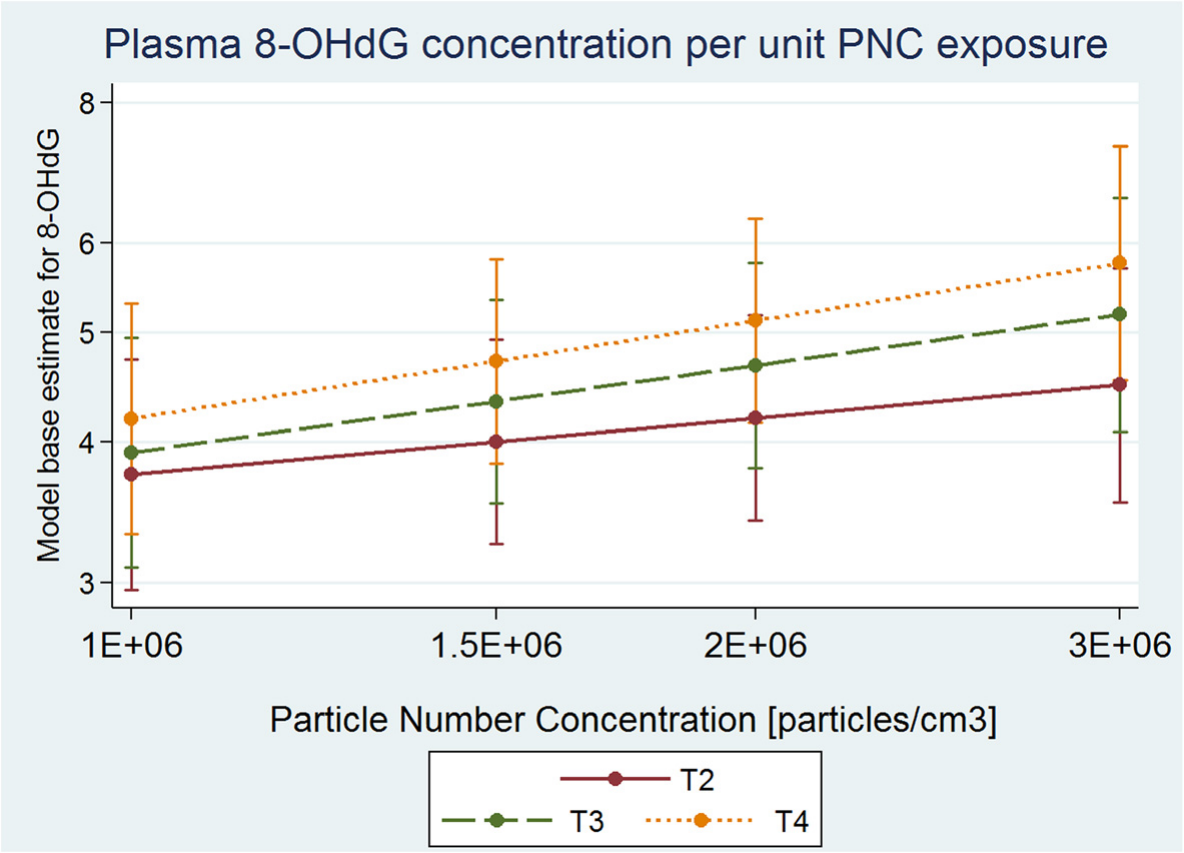
Plasma-8-OHdG concentration per unit PNC exposure. Linear mixed model based estimate for T2, T3, T4 plasma-8-OHdG concentrations presented by increasing per unit particle number concentration (PNC)

Plasma-8-OHdG concentrations increased between T1-T4 by 26 % ([95 % CI: 8 %; 47 %]) in the high PNC exposure group (PNC ˃1.6E + 06 particles/cm^3^; *N* = 11), which was significantly higher (*p* = 0.03) than the T1-T4 increase of 2.1 % ([95 % CI: -14 %; 21 %]) in the low PNC exposure group (PNC ˂1.6E + 06 particles/cm^3^; *N* = 9) (Fig. 2). Results of the mixed model to account for quantitative trends between PNC exposure and plasma-8-OHdG concentrations showed that there was a significant increase in plasma-8-OHdG concentrations per unit exposure of PNC from T1 to T3 (0.591 ± 0.25, *p* = 0.02) and from T1 to T4 (0.668 ± 0.25, *p* = 0.01). These results indicate that doubling exposure to PNC would induce a 19 % significant increase in plasma-8-OHdG concentrations at T3 (*p* = 0.02) and 22 % significant increase in plasma-8-OHdG concentrations at T4 (*p* = 0.01). Figure 3 presents the slopes of plasma-8-OHdG concentration due to PNC exposure at different time points, and shows that as exposure to PNC increases, the plasma-8-OHdG concentration increases.

#### Particle size

Urinary-8-OHdG concentrations increased from T1 to T4 by 74.5 % ([95 % CI: 48.4 %; 100.5 %]) for the group exposed to particles with smaller GMDs (GMD<44 nm; *N* = 10), and by 25.6 % ([95 % CI:1.6 %; 49.6 %]) in the group exposed to particles with larger GMDs. However, the difference in increase between the two groups was not significant. In plasma, 8-OHdG concentrations increased from T1-T4 by 19.5 % ([95 % CI: 11 %; 28 %]) in volunteers exposed to particles with smaller GMDs, which was significantly higher (*P* = 0.03) than the 9.6 % ([95 % CI: 1.1 %; 18.1 %]) increase for volunteers exposed to particles with larger GMDs.

#### ROS production potential

Plasma-H_2_O_2_ concentrations increased from T1 to T4 by 28 % ([95 % CI: 2 %; 60 %]) in the high ROS production potential group (>10 nmol/m^3^; *N* = 8), and by 22 % ([95 % CI:1 %; 50 %]) in the low ROS production potential group (<10 nmol/m^3^; *N* = 12). However, the difference between the two ROS-potential groups was not significant.

#### Gravimetric mass

We also did not see any significant associations between gravimetric mass exposure variables and oxidative stress biomarker concentrations.

#### Welding technique

We also assessed biomarker time course changes by welding technique in regards to burning (i.e., oxidation) of the welding task by the volunteer. We found that the increase between T1-T4 for urinary-8-OHdG was 74.9 % ([95 % CI: 52.3 %; 96.5 %]) for the group that did not burn their welding task (*N* = 12), which was significantly higher (*p* = 0.01) when compared with the T1-T4 increase of 8.9 % ([95 % CI: -18.2 %; 36 %]) of the group that burned their welding task (*N* = 8).

#### Evolution of total reducing capacity

There was no correlation between the total reducing capacity concentrations, an indicator for antioxidants, and any of the exposure variables. An assessment of the effects of exposure on the ratio of H_2_O_2_/total reducing capacity, which could be interpreted as the oxidative balance, showed that there was no correlation between exposure variables and the proposed oxidative balance.

The increase of urinary-H_2_O_2_ concentrations was not significantly associated with any of the exposure variables.

### Self reported health data

Throat itchiness/discomfort, difficulty breathing, and fatigue were the most often reported symptoms on exposure days. On exposure days, 55 % of volunteers reported some indication of throat itchiness/discomfort, 70 % reported some indication of difficulty breathing; and 70 % reported fatigue. Sign rank results show that indications of eye discomfort, throat itchiness/discomfort, shortness of breath, cough, chest tightness, headache, and fatigue were significantly higher when compared with control days. Self reported irritating symptoms scores were not correlated with exposure variables nor with oxidative stress biomarkers.

## Discussion

Results of this study show that a 60-min exposure to TIG welding fume was associated with a significant increase of plasma and urinary-H_2_O_2_ concentrations, and plasma and urinary-8-OHdG concentrations at 3 h post exposure in apprentice welders. Our findings are the first to show that short-term exposure to TIG welding fume, even in well controlled and ventilated environments, may result in acute oxidative stress reactions in healthy, non-smoking individuals not chronically exposed to welding fume. Our results also provide evidence that PNC and particle size may be associated with the acute increase of plasma-8-OHdG concentrations.

### Increase in oxidative stress

Our oxidative stress results confirm what has been reported in previously published literature, in that welding fume exposure may result in systemic oxidative stress, both in rodents [17] and in humans [2, 16, 44]. Erdely et al. [17] found in rats an increase in mononuclear cell oxidative stress in circulating blood cells following the pulmonary instillation of metal-rich particulate matter from manual metal arc welding. du Plessis et al. [16] also found increased oxidative stress in circulating mononuclear cells in male welders (*n* = 15) occupationally exposed to welding fumes. Our study is unique in that it specifically investigates aluminium TIG welding fume, which has been previously shown to produce a large majority at the nanoscale and have a low agglomeration rate [5, 6, 23, 54].

Mean basal plasma-H_2_O_2_ levels in our study (5.0 ± 2.4 μM) were similar to what has been previously reported by [13] (6.3 ± 1.1 μM), and higher than what has been previously reported by Lacy et al. [37] (2.14 ± 0.13 μM). Other studies have shown that plasma-H_2_O_2_ basal values can be variable in healthy subjects, with values ranging from close to zero [21] to as high as 35 μM [66]. In human plasma, H_2_O_2_ is known to diffuse into circulating blood cells and platelets for metabolism [25]. The acute increase in H_2_O_2_ concentrations reported in our study may be in part related to its ability to diffuse rapidly and readily within and between cells [4, 25]. Mean basal urinary-H_2_O_2_ values in our study were higher than previously reported in healthy volunteers (uncorrected: 36.6 ± 38.8 μM versus a range of 11.7-18.4 μM as reported by [46]). However, other studies have demonstrated that at baseline conditions, urinary H_2_O_2_ can be very high, even exceeding 100 μM [25]. Moreover, urinary- H_2_O_2_ concentrations in healthy volunteers demonstrate significant variability [3], and our uncorrected mean values and high standard deviation support these findings. Urinary H_2_O_2_ excretion is a human metabolic mechanism for controlling levels under conditions of oxidative stress. As such, our data confirms that excretion of H_2_O_2_ was efficient as the associated concentration increase was found to be the largest in urine.

The acute increase in plasma and urinary-8-OHdG concentrations in this study are interesting to consider given the rather complex mechanisms related to the production of this molecule. One of the most sensitive and most widely measured biomarkers of oxidative DNA damage is 8-OHdG [26, 29, 48, 65]. Specifically, it is a known biomarker of OH•-mediated DNA lesions [48] and has been used to measure DNA damage in humans exposed to a wide range of toxicants. While other DNA nucleobases may react with OH•-, 8-OHdG represents the most abundant DNA lesion due to its relative ease of formation and its promutagenic nature [65]. Experimental data suggests that the presence of 8-OHdG in the human body most likely results from the oxidation of the deoxynucleotide pool and does not represent solely repairing/excretion of the oxidized DNA guanine [40]. Once generated, 8-OHdG has been shown to be very stable, and does not undergo metabolization processes in human circulation [11]. Mean basal levels of plasma-8OHdG in our study (4.4 ± 2.4 μg/l) were higher than what has been previously reported by Sato et al. [57]: 0.3 ± 0.1 μg/l in control volunteers, and by Lee et al. [40]: 2.2 ± 0.1 μg/l in non-smoking controls. Mean basal levels of urinary-8OHdG (3.0 ± 5.6 μg/g creatinine) were in agreement with previously reported baseline values by Sauvain et al. [58]: 0.34-7.21 μg/g creatinine; median 2.46 μg/g creatinine for non-smokers even though we used a different assay to quantify 8-OHdG. In regards to its time course changes, the significant increase in urinary-8-OHdG concentrations at 3 h post exposure reported in our study is in agreement with studies that have shown rapid urinary excretion of 8-OHdG (i.e., within 24 h) following occupational exposure to potentially oxidizing agents [53]. For the biomarkers with significant increases from T1-T4 (plasma and urinary-H_2_O_2_ and plasma and urinary-8-OHdG), a temporal increase in coefficient value matched with decreasing *p*-value (Additional file 4: Table S4) is observed over the time points (i.e., coefficient values T1-T2 < T1-T3 < T1-T4). This trend is particularly important for the increase of plasma-8-OHdG concentrations, which showed a larger increase from T1-T4 (22 %, *p* = 0.01) than from T1-T3 (19 %; *p* = 0.02) as a function of per-unit PNC exposure. Results confirm that 3 h post exposure marked the most important time point for biomarker increase within the framework of our study. However, we did not witness any indication of a peak and gradual decrease of biomarker concentrations to control-day levels. This may be the result of the short time frame considered, which presents a limitation in this study for the analysis of extended biomarker kinetics (i.e., >3 h post exposure). It is recommended that future studies include additional time points (e.g., 12 h and 24 h post exposure) for more comprehensive investigation into plasma and urinary H_2_O_2_ and 8-OHdG time course changes, particularly as a function of PNC exposure.

We observed significant changes in oxidative stress biomarkers concentrations in plasma and urine, but not in collected EBC. This observation is in agreement with previous occupational health studies, most of which also found little or no signs of oxidative stress or inflammation from EBC biomarkers after exposure. Brand et al. [6] studied the effects of 6-h metal active gas welding (MAG) exposure on healthy young male subjects and found no changes in inflammatory biomarkers detected from EBC. While Laumbach et al. [39] observed a slight increase (10 %) in EBC MDA concentration after exposure to traffic air pollutants, the results were not statistically significant. Similarly, Riddervold et al. [55] examined the effects of wood smoke exposure and also reported limited signs of inflammation from EBC samples. In the collected EBC of retired miners with chronic obstructive pulmonary disease (COPD), there was a non-significant correlation between EBC H_2_O_2_ concentration with COPD [41]. The few studies that have shown significant changes in EBC biomarker concentrations focused on subjects with severe and chronic disease state, or subjects exposed to elevated levels of known toxic substances. For example, Chan et al. [8] found that lung cancer patients had significantly higher levels of H_2_O_2_ concentrations in their EBC compared to control subjects, while Kostikas et al. [35] found that one-hour inhalation of second hand smoke, a known inducer of oxidative stress, caused significant H_2_O_2_ increase in collected EBC from healthy non-smokers. It is thus not entirely surprising that we did not observe any significant increase in oxidative stress biomarkers in the collected EBC. It is worth noting that EBC analysis currently lacks standardization and faces many methodological limitations, which also makes it difficult to compare reference levels from the literature. Moreover, EBC is mostly compromised of water and may also include droplets of respiratory tract lining fluid (RTLF) that are released from the surfaces of the airway. A limitation of EBC analysis for oxidative stress markers is the large dilution of the RTLF, resulting in low concentrations of EBC constituents with detection levels that are often close the limits of analytical methods [14]. The large number of EBC biomarker measures (49 % EBC-H_2_O_2_; 28 % EBC-MDA) that were below the LOD presented a significant limitation for analysis in our study. In particular, the FOX assay technique used to assess EBC-H_2_O_2_ faces limitations due to the interference of antioxidants that may reduce the Fe(III) to Fe(II) with a concomitant loss of absorbance, thereby resulting in negative values. It is also noteworthy that the volunteers in our study were healthy young non-smokers, which is in contrast to those studies that looked at older workers chronically exposed to welding fumes [6].

### Mechanisms of oxidative stress

An important question regarding the oxidative stress is its localisation. We previously proposed that this stress starts at the moment the particle deposits in the alveolar space, resulting in high local ROS-levels around the point of impact, which then induce responses in adjacent cells and in the capillary endothelium which is in close proximity (i.e., distance of a few hundred nm), resulting in a cascade of both local and systemic response [56]. Our controlled human exposure experiments were conducted with healthy teenage apprentices, whose average age was 17 years. For ethical reasons, less-invasive biofluid collection methods were chosen over invasive medical procedures, such as bronchial alveolar lavage or lung biopsies. As a consequence, it was not possible to examine the mechanistic details of oxidative stress response on an organ or cellular level. However, the collected data does not disagree with the hypothesis of oxidative stress generation as largely occurring in the alveolar space. Welding fume PM contains transition metals known to catalyze redox reactions in the lung by inducing cyclical chemical reactions resulting in production of hydroxyl radicals, superoxide anion, singlet oxygen, and hydrogen peroxide [15, 33, 50, 60, 63]. H_2_O_2_ in EBC was in many cases below detection limit, but higher than what would be expected if it simply corresponded to H_2_O_2_ levels measured in plasma. H_2_O_2_ can also diffuse rapidly and readily between and within cells, which enhances the cascade of systemic oxidant response [4, 25]. In regards to the significant increase witnessed for 8-OHdG, welding fume PM metal components are known to promote the oxidation of nucleobases and the intracellular nucleotide pool [33, 52]. The mechanism by which the metal component of welding fumes can cause oxidative stress may act not only by directly producing radicals but also through signalling pathways, such as via mitogen-activated protein kinases (MAPK) and NF-kB, to generate pro-inflammatory mediators that results in a state of inflammation [15, 50]. While the assessment of pro-inflammatory mediators is beyond the scope of this study, they merit further attention in future studies of human exposures to TIG welding fume.

### Oxidative stress and exposure variables

We observed a significantly larger effect in the increase of plasma-8-OHdG concentrations for apprentices exposed to high PNC (>1.6E + 06 particles/cm^3^). Increased plasma-8-OHdG concentrations were associated with a per unit increase of PNC, suggesting a potential exposure-response relationship. Moreover, there was a significantly larger effect in the increase of plasma-8-OHdG concentrations for apprentices exposed to smaller welding fume particles (GMD˂44 nm). Fine particulate matter (<2.5 μm) has been shown to promote the formation of 8-OHdG in vitro [59], and several human studies that have investigated particulate exposures and 8-OHdG concentrations provide evidence of an association, and even preliminary exposure-response metrics [53]. Sorensen et al. [62] measured personal PM_2.5_ exposure in 50 healthy students and found that personal exposure to PM_2.5_ was a predictor of 8-OHdG in lymphocyte DNA with an 11 % increase of 8-OHdG per μg/m^3^ increase in personal PM_2.5_ exposure. Kim et al. [34] investigated the urinary 8-OHdG concentrations of boilermakers (*N* = 20) exposed to fine particles from welding fumes and residual oil fly ash, and found a significant increase in urinary-8-OHdG levels from pre-shift to post-shift when adjusting for smoking and age. The authors found a positive exposure–response relationship between urinary-8-OHdG concentrations and PM_2.5_ exposure. Another study assessed 41 workers who conducted 6 h of stick welding with mild steel, and found a significant pre- to post-shift increase in urinary-8-OHdG concentrations. Exposure to PM_2.5_ was a significant predictor of urinary-8-OHdG concentrations [51].

It has been shown that there is a strong correlation between PNC and active particle surface area in the presence of high concentrations of ultrafine particles, particularly in occupational settings dominated by combustion particles [27]. Surface area characteristics are important indicators of potential toxicity of NPs, and the presence of functional groups adsorbed on particles, such as metal ions and organics has been found to generate ROS leading to oxidative stress in biological systems [49]. As such, the active surface area of TIG welding fume NPs may favor the creation of functional groups, in turn increasing the probability of ROS generation.

Our findings of increased plasma-8OHdG concentration with per unit PNC exposure increase, yet no associations of biomarker concentrations with gravimetric mass, question the applicability of gravimetric mass metrics as occupational exposure limits for welding tasks. The gravimetric mass of TIG welding fume particles is much lower compared to that of welding processes that produce larger particles, such as metal inert gas (MIG) or metal active gas (MAG) [5, 42]. As such, PNC may be a better predictor of risks related to oxidative stress response than gravimetric mass for inhaled particles in the nanoscale. However, it is important to note that in our exposure characterization, we witnessed much higher levels of PNC than gravimetric mass due to the negligible weight of TIG welding fume NPs. Thus uncertainty estimates for mass may be much larger when compared with PNC. As such, we cannot exclude that our lack of correlation between mass and oxidative stress biomarker increase was due to much larger uncertainty estimate for mass than PNC due to the method of measurement. Despite this limitation, it may be the case that initiatives that aim to reduce gravimetric mass may not be effective in decreasing the level of risk in TIG welding settings.

Results from our mixed model suggest that doubling PNC may result in a significant 22 % increase in plasma-8-OHdG concentrations. Given the realities of welding workplaces that may lack appropriate (or any) ventilation, and may be contaminated by other welding or brazing aerosols, it is not difficult to imagine that PNC exposures may be much higher than in our controlled, experimental setting. Such realities are made more hazardous if workers lack appropriate respiratory protection. As 8-OHdG represents a pre-mutagenic DNA adduct that has been implicated in carcinogenesis [48], it is critical that exposures to elevated PNC in TIG welding are reduced towards the aim of better health and safety of welders worldwide. This finding has important implications for young workers who may face chronic exposures throughout a lifetime of work in the welding industry. Moreover, as TIG welding fume is generally less visible than other welding fume when produced, assumptions amongst welding trainers exist that TIG welding is cleaner and less dangerous ([5]; Personal communication with welding trainers of the Center for Professional Training, Lausanne, Switzerland, January 2014). Findings of this study highlight the importance of increasing awareness on TIG welding fume toxicity. It is also recommended that in addition to PNC, deposited dose of inhaled TIG welding fume NPs is assessed to provide information on the fate of these particles in the human respiratory system towards more precise occupational risk assessments [22].

## Conclusion

In this study we assessed the effect of TIG welding fume exposure on the time course changes of multiple oxidative stress biomarkers in the EBC, blood, and urine of apprentice welders before, and at several time points after exposure. We found that a single 60-min exposure to TIG welding fume was associated with a significant increase in plasma and urinary-H_2_O_2_ concentrations, and plasma and urinary-8-OHdG concentrations at 3 h post-exposure. Our findings are the first to show that short-term exposures to TIG welding fume, even in controlled and well-ventilated settings, may result in acute oxidative stress reactions in healthy, non-smoking individuals not chronically exposed to welding fumes. We also found that PNC, but not gravimetric mass, was associated with the oxidative stress biomarkers measured in plasma, providing a consideration for the use of additional metrics in occupational exposure assessments for TIG welding workplaces. Given the realities of welding settings that may lack appropriate ventilation, and beliefs among welders that TIG represents a cleaner and safer welding process, it is recommended that the awareness concerning the potential toxicity of TIG welding fumes is increased among welding populations.

## Methods

### Study participants and study design

We recruited participants by visiting local apprentice welding schools in Southwestern Switzerland. Participants were nonsmokers, 16-25 years of age, male, without history of respiratory diseases or conditions, cardiovascular difficulties, not taking medications linked to cardiac, respiratory diseases, or any medications that can modify dilation, not taking drugs or alcohol and not excessively exposed to fine particles during work outside of apprentice training. All participants provided informed written consent prior to participating in the study. The study was approved by the Ethics Committee of Canton de Vaud, Lausanne, Switzerland (Study Protocol No. 389/13), and was conducted in accordance with the Declaration of Helsinki. A pilot study was conducted with four volunteers between January and February 2014 to validate study methodology and to interview welding trainers on risk perception of TIG welding. The full study was conducted between March and November 2014.

The study design was chosen to be able to a) assess the acute time course changes of oxidative stress biomarkers over a series of time points and b) to match each volunteer exposure results with their own control results. As such, each volunteer participated in two study days: a control day in which they were exposed to high efficiency particulate absorption (HEPA)-filtered air in an exposure cabin for 60 min; and an exposure day in which they were exposed to TIG welding fume in an exposure cabin for 60 min. On both study days, EBC, blood and urine were collected before exposure (8:30 h, T1); immediately after exposure (10:00 h, T2); 1 h after end of exposure (11:00 h, T3); and at 3 h after end of exposure (13:00 h, T4) (See Additional file 5: Figure S1).

To control for fluctuations in biomarkers due to circadian rhythm, both study days started in the morning and collected biological liquids following the same above-mentioned schedule. On the morning of study days volunteers were asked to avoid all types of caffeinated food and drinks, and particularly coffee as it has been shown to artificially raise urinary H_2_O_2_ levels ([47]; [28]. Volunteers were also instructed to avoid fruits and vegetables (including juices) that contain ascorbic acid due to its potential influence on total reducing capacity measures. Volunteers were instructed to consume a light breakfast, which was to be the same on both control and exposure study days to avoid influence on oxidative status from diet. A self-reported health questionnaire was given before (T1) and after (T2) welding fume exposure or controlled air exposure. The questionnaire at T1 asked about volunteers’ dietary intake in the last 24 h to account for potential interactions with oxidative status. Volunteers were instructed to wear a valved, disposable respirator (Aura 9332, 3 M) to the study day in order to avoid outdoor particulate exposures.

### Welding fume exposure

Volunteers were asked to conduct a 60 min TIG welding task with aluminium OK tigrods (ESAB, 4043, diameter 2.4 mm) on aluminium cubes (12 cm × 12 cm × 12 cm). This welding task represents a standardized exercise performed in welding schools across Western Switzerland. The aluminium OK tigrods were composed of >92 % of Al; 4.5–6 % Si; 0.8 % Fe; 0.5 % Mn; 0.5 % Mg; 0.3 % Cu; 0.2 % Ti; 0.1 % Zn and the aluminum cube metal was composed of >97 % of Al; 0.7 % Fe; 0.5–1.1 % Mg; 0.3 % Si; 0.25 % Zn; 0.2 % Mn; 0.2 % Cu; 0.1 % Cr. TIG welding was done using an ESAB CaddyTig 2200i AC/DC machine (Stucki Soudure SA, Switzerland) supplied with a 98 % W, 2 % Ce electrode and 100 % argon shield gas. Volunteers wore a non-ventilated welding helmet with auto-darkening lens. The welding task was conducted in a 10 m^3^ exposure cabin with a controlled pulsing ventilation system, an exchange rate of 9.3 h^−1^ and a high efficiency particulate absorption (HEPA) filter for the incoming and outgoing air [24].

### Experimental set up, sampling and characterisation of samples

Details of the welding station set up, sampling and characterisation of samples has been fully described in [23]. Briefly, volunteers were equipped with personal sampling monitors that were attached to the inside of the customized welding helmet, and included a MiniParticle Sampler (INERIS, France) with a copper mesh Transmission Electron Microscopy (TEM) grid attached to an Escort Elf personal pump operating at 0.3 L/min; a PM_4_ Parallel Particle Impactor (PPI, SKC Inc., USA) containing a 37 mm PTFE filter (Pall Life Sciences, USA), attached to a Leland Legacy personal pump (SKC Inc., USA) with flow rate of 8 L/min; and the inlet impactor (0.8-μm cut-off) of a DiscMini particle counter (Matter Aerosol, Switzerland). As part of the exposure characterization, we assessed particle number concentration, geometric mean diameter, particle morphology, gravimetric mass, elemental composition, gaseous components, and acellular ROS generation.

### Collection and preservation of biological liquids

Prior to EBC collection, volunteers rinsed out their mouth with water to avoid contamination. A one-time use Rtube (Respiratory Research, Atlanta, USA) was removed from its plastic wrapper and inserted into an Rtube cooling sleeve that had been cooled to −25 °C. Volunteers were asked to wrap their lips around the breathing zone and to breathe normally for 10 min into the Rtube while sitting and wearing a nose clip. Up to 2 mL of EBC were recovered and weighed. Approximately 50 μL of EBC were used immediately to assess total reducing capacity. The remaining EBC was aliquoted and flash frozen. EBC samples were stored at −70 °C until analysis.

Approximately 6 mL of blood was collected by venipucture into vacutainer blood collection tubes containing lithium-heparin (BD, USA). Approximately 50 μL were used immediately to assess total reducing capacity. The remaining blood was centrifuged and the plasma was aliquoted and flash frozen. Plasma samples were stored at − 70 °C until analysis. Spot urine samples were collected in sterile cups from volunteers and approximately 50 μL were used immediately to assess total reducing capacity. The remaining urine was aliquoted and flash frozen. Creatinine was determined following the Jaffe method. The remaining urine samples were stored at −70 °C until analysis.

### Measurement of biomarkers in biological liquids

#### Total reducing capacity

The total levels of reducing species were measured with a redox sensor that is based on an electrochemical technique and responds to all water-soluble compounds in biological fluids that can be oxidized within a defined electrochemical potential range (Edel therapeutics SA, Lausanne, Switzerland). Total reducing capacity, as measured by this method, corresponds to a defense against oxidative stress, thus providing a potential indicator of antioxidant action [45]. The redox sensor requires ionic capacity in analyzed matrices. Diluted EBC, fresh whole blood and fresh spot urine were analyzed immediately after collection by loading 10 μL onto the EDEL sensor chip and inserting it into the EDELSCAN device, as previously described by [58]. As EBC is composed of mostly water, the collected EBC sample was diluted 2 times in phosphate buffer (pH 7.4, 0.1 M) before analysis to ensure adequate ionic strength. Fresh, whole blood was analyzed immediately and was not pre-treated nor conditioned to assess the ability of the sample as a whole to donate electrons [64]. It is worthwhile to note that according to the manufacturer, iron does not specifically interfere with the electrochemical measurement. Three replicates were conducted for each sample.

#### Hydrogen peroxide

H_2_O_2_ concentrations were analyzed in EBC, plasma, and urine using the validated ferrous-ion oxidation-xylenol orange assay (FOX) [46]. The FOX assay technique is based on the oxidation of iron, which can be achieved by various peroxides. As the calibration was done with H_2_O_2_, the obtained results are expressed as H_2_O_2_ equivalent. Three replicates were taken for each sample. A prepurification step removing biological components larger than 10,000 dalton was done for H_2_O_2_ analysis in plasma and the standard addition technique was used for quantification in plasma and urine. The averaged LOD, corresponding to three times the standard deviation of the blank in the three biological matrices is given in Additional file 2: Table S1.

#### 8-OHdG

8-OHdG levels were measured in plasma and urine using a commercially available enzyme-linked immunosorbent assays (ELISA) (Japan Institute for the Control of Aging, Shizuoka, Japan). The selected kit uses an anti 8-OHdG monoclonal antibody (clone N45.1) which is highly specific for quantifying the oxidative DNA adduct 8-OHdG in human tissue, serum, plasma, and urine. This method yields results that are well correlated with results from high-performance liquid chromatography (HPLC) and HPLC- Electrochemical detection (ECD) [19, 61, 67]. Three replicates were conducted for each sample.

#### MDA

MDA was analyzed in EBC with a validated method which uses derivatization of the analyte with thiobarbituric acid, followed by high-performance liquid chromatography (HPLC) separation and fluorescence detection [38]. This method is highly sensitive for EBC, but is prone to interferences and the presence of artifacts when used for plasma or urine analysis [12, 32]. In order to avoid such interferences, a different and more appropriate method for analyzing MDA in plasma and urine was selected. Free MDA in plasma and urine was derived with pentafluorophenylhydrazine, isolated from the matrix and analyzed by gas chromatography–mass spectrometry (GC-MS) [10]. Two replicates were conducted for each sample. The averaged LOD, corresponding to three times the standard deviation of the blank in the three biological matrices is given in Additional file 2: Table S1.

### Questionnaires

After exposure to either HEPA-filtered air on control days or TIG welding fumes on exposure days, volunteers were asked to complete a likert-scale questionnaire regarding 11 self-perceived health symptoms [18] were asked to rank their symptoms of eye irritation, nose irritation, throat discomfort, difficulty breathing, cough, chest tightness, headache, fatigue, nausea, feeling of intoxication, and dizziness.

### Data treatment and statistical analysis

Statistical analyses were performed using STATA 13 (StataCorp LP, College Station, TX, USA).

Concentrations of H_2_O_2_, MDA, 8-OHdG and reducing capacity were log-transformed to normalize their distribution. The evolution of log(reducing capacity), log(H_2_O_2_), log(MDA), log(8-OHdG) was analyzed using a linear mixed model with the subject considered as a random effect and considering within-day (between time points) and between-day (control and exposure day) differences as main independent effects. We applied a linear mixed model to assess associations with exposure variables for each volunteer (particle number concentration, gravimetric mass, geometric mean diameter, and acellular ROS production potential). To assess exposure variable interactions with biomarker concentrations more precisely, we divided volunteers in two exposure groups based on median exposure values to ROS production potential (low and high ROS); PNC exposure (low and high PNC); and particle GMD (smaller and larger GMD). We also divided the volunteers into two groups based on their welding technique in regards to oxidation burn marks on the aluminium cube. Evidence of these burn marks allowed separating the volunteers into two distinct groups based on welding performance: one without any evident burns on their cube (*N* = 12) and one with significant burn marks (*N* = 8) (see [23] for full results). A sign rank test was applied to assess the ordinal differences for self-reported health symptoms for the control and exposure day. Interactions were explored between and within-day differences. Residual plots allowed the identification of potential outliers, which were tentatively excluded in subsequent analyses to assess the robustness of the results. Bonferroni method was applied to correct for correlations within positively dependent indicators. Significance was set at <0.05 level.

## Abbreviations

8-OHdG, 8-hydroxy-2′-deoxyguanosine; BZ, breathing zone; DCFH, 2′,7′-dichlorofluorescin; EBC, exhaled breath condensate; GMD, geometric mean diameter; H_2_O_2_, hydrogen peroxide; MDA, malondialdehyde; NF, near-field; NP, nanoparticle; PNC, particle number concentration; ROS, reactive oxygen species; TIG, tungsten inert gas

## Additional files

Additional file 1: Table S3. Control day: Median concentrations of oxidative stress biomarkers in EBC, plasma and creatinine corrected urine (respective unit/g creatinine), presented by time point. EBC was not assessed for 8-OHdG concentrations. ^+^Total reducing capacity concentration was measured in whole blood and not plasma. (DOC 47 kb)

Additional file 2: Table S1. Percentage of samples with concentrations below the Limit of Detection (LOD) for biomarkers in biological liquids, in total. ^+^Total reducing capacity concentration was measured in whole blood and not plasma. (DOC 29 kb)

Additional file 3: Table S2. Percentage of samples with concentrations below the limits of Detection (LOD) for MDA and H_2_O_2_ concentrations in EBC, shown by study day and timepoint. (DOC 35 kb)

Additional file 4: Table S4. Exposure day: complementary table to main results, Table 4. Coefficients with standard error and *p*-value for the different mixed models used for explaining the evolution of oxidative stress biomarkers in EBC, plasma and creatinine-corrected urine. *Indicates significant increase at the time point as compared to T1. ^+^Total reducing capacity concentration was measured in whole blood and not plasma. (DOC 47 kb)

Additional file 5: Figure S1. Schematic of exposure day sample collection and questionnaire schedule. Control day follows same schedule, but replaces welding fume exposure with HEPA-filtered air exposure. (DOC 41 kb)
