# Supplementary material for: Increase in oxidative stress levels following welding fume inhalation: a controlled human exposure study
Source: Part Fibre Toxicol. 2016 Jun 10;13:31. doi: 10.1186/s12989-016-0143-7 (PMC4901438; doi:10.1186/s12989-016-0143-7)
Supplement: Supplementary file 1 — Control day: Median concentrations of oxidative stress biomarkers in EBC, plasma and creatinine corrected urine (respective unit/g creatinine), presented by time point. EBC was not assessed for 8-OHdG concentrations. +Total reducing capacity concentration was measured in whole blood and not plasma. (DOC 47 kb) [file 12989_2016_143_MOESM1_ESM.doc]

Additional file 1: Table S3. Control day: Median concentrations of oxidative stress biomarkers in EBC, plasma and creatinine corrected urine (respective unit/g creatinine), presented by time point. EBC was not assessed for 8-OHdG concentrations. ^+^Total reducing capacity concentration was measured in whole blood and not plasma.

|  |  | **EBC** | **PLASMA** | **CREATININE-CORRECTED URINE** |
| --- | --- | --- | --- | --- |
|  | Time | Median (range) | Mean (range) | Mean (range) |
| **Total Reducing Capacity [a.u.]** | 1 | 27.4 (20-38.5) | 161.0 (117.7-212.7)^+^ | 1831.8 (938.4-7766.7) |
|  | 2 | 27.3 (16.7- 44.5) | 164.0 (120.5-216.3)^+^ | 2574.2 (1520.8-7332.9) |
|  | 3 | 27.4 (18- 45.5) | 162.8 (106.5-224)^+^ | 2314.8 (1282.3- 3587.7) |
|  | 4 | . 27.5 (17.3- 43) | 161.3 (113.5-217.7)^+^ | 2485.5 (465.6- 3698.5) |
| **H_2_O_2_ [µM]** | 1 | 0.05 (˂LOD-0.50) | 4.47 (1.4-9.5) | 24.9 (2.0-507.5) |
|  | 2 | 0.04 (˂LOD-1.0) | 4.5 (1.4-13.5) | 23.29 (3.0- 809.5) |
|  | 3 | 0.08 (˂LOD-0.7) | 4.37 (˂LOD -19.4) | 26.6 (6.1-99.0) |
|  | 4 | 0.08 (˂LOD-0.4) | 4.5 (1.4-8.0) | 27.0 (3.3-121.5) |
| **MDA [nM]** | 1 | 5.1 (˂LOD-14.3 | 121.9 (˂LOD-190.3) | 236.0 (127.6-775.5) |
|  | 2 | 6. 2 (˂LOD-21.6) | 113.3 (˂LOD - 202.9) | 233.5 (131.3-557.1) |
|  | 3 | 6.2 (˂LOD- 17.5) | 113.0 (˂LOD - 208.5) | 207.5 (131.0- 564.3) |
|  | 4 | 5.8 (˂LOD-30.6) | 117.9 (˂LOD - 394.1) | 193.6 (63.6-450) |
| **8-OHdG [μg/l]** | 1 | - | 3.4 (˂LOD-13.6) | 1.7 (˂LOD -12.1) |
|  | 2 | - | 3.6 ˂LOD -11.0) | 1.7 (˂LOD -29.8) |
|  | 3 | - | 4.2 (˂LOD -17.0) | 1.4 (˂LOD -21.5) |
|  | 4 | - | 3.9 (˂LOD -20.0) | 1.7 (˂LOD -34.7) |
