# Supplementary material for: Increase in oxidative stress levels following welding fume inhalation: a controlled human exposure study
Source: Part Fibre Toxicol. 2016 Jun 10;13:31. doi: 10.1186/s12989-016-0143-7 (PMC4901438; doi:10.1186/s12989-016-0143-7)
Supplement: Supplementary file 2 — Percentage of samples with concentrations below the Limit of Detection (LOD) for biomarkers in biological liquids, in total. +Total reducing capacity concentration was measured in whole blood and not plasma. (DOC 29 kb) [file 12989_2016_143_MOESM2_ESM.doc]

Additional file 2: Table S1. Percentage of samples with concentrations below the Limit of Detection (LOD) for biomarkers in biological liquids, in total. ^+^Total reducing capacity concentration was measured in whole blood and not plasma.

|  | **EBC** | **Plasma** | **Urine** |
| --- | --- | --- | --- |
| **Total Reducing Capacity** | 0% | 0%^+^ | 0% |
| **H_2_O_2_** | 48.8% | 0.002% | 0% |
| **MDA** | 27.8% | 1.3% | 0% |
| **8-OHdG** | - | 0.2% | 0.4% |
