# Supplementary material for: Increase in oxidative stress levels following welding fume inhalation: a controlled human exposure study
Source: Part Fibre Toxicol. 2016 Jun 10;13:31. doi: 10.1186/s12989-016-0143-7 (PMC4901438; doi:10.1186/s12989-016-0143-7)
Supplement: Supplementary file 3 — Percentage of samples with concentrations below the limits of Detection (LOD) for MDA and H2O2 concentrations in EBC, shown by study day and timepoint. (DOC 35 kb) [file 12989_2016_143_MOESM3_ESM.doc]

Additional file 3: Table S2. Percentage of samples with concentrations below the limits of Detection (LOD) for MDA and H_2_O_2_ concentrations in EBC, shown by study day and timepoint.

|  | **MDA in EBC** | **H_2_O_2_ in EBC** |
| --- | --- | --- |
| **Total** | 27.8% | 48.8% |
| **T1 (total)** | 7.8% | 13.6% |
| **T2 (total)** | 5.3% | 13.3% |
| **T3 (total)** | 6.8% | 11% |
| **T4 (total)** | 8.5% | 10.9% |
| **Control days (total)** | 13.5% | 20% |
| **Control T1** | 3.7% | 6% |
| **Control T2** | 2.6% | 6% |
| **Control T3** | 4.2% | 4% |
| **Control T4** | 4.2% | 4% |
| **Exposure days (total)** | 14.3% | 28.8% |
| **Exposure T1** | 4.1% | 7.6% |
| **Exposure T2** | 2.6% | 7.3% |
| **Exposure T3** | 3.3% | 7% |
| **Exposure T4** | 4.2% | 6.9% |
