# Supplementary material for: Increase in oxidative stress levels following welding fume inhalation: a controlled human exposure study
Source: Part Fibre Toxicol. 2016 Jun 10;13:31. doi: 10.1186/s12989-016-0143-7 (PMC4901438; doi:10.1186/s12989-016-0143-7)
Supplement: Supplementary file 4 — Exposure day: complementary table to main results, Table 4. Coefficients with standard error and p-value for the different mixed models used for explaining the evolution of oxidative stress biomarkers in EBC, plasma and creatinine-corrected urine. *Indicates significant increase at the time point as compared to T1. +Total reducing capacity concentration was measured in whole blood and not plasma. (DOC 47 kb) [file 12989_2016_143_MOESM4_ESM.doc]

Additional file 4: Table S4. Exposure day: complementary table to main results, table 4. Coefficients with standard error and p-value for the different mixed models used for explaining the evolution of oxidative stress biomarkers in EBC, plasma and creatinine-corrected urine. *Indicates significant increase at the time point as compared to T1. ^+^Total reducing capacity concentration was measured in whole blood and not plasma.

|  |  | **EBC** | **PLASMA** | **CREATININE-CORRECTED URINE** |
| --- | --- | --- | --- | --- |
|  | Time | Coefficients with standard error  (p-value) | Coefficients with standard error  (p-value) | Coefficients with standard error (p-value) |
| **Log-Total Reducing Capacity** **[a.u.]** | T1 | - | - | - |
|  | T2 | -0.013 ± 0.096 (0.893) | -0.047 ± 0.046 (0.314) ^+^ | -0.038 ± 0.122 (0.752) |
|  | T3 | 0.046 ± 0.096 (0.632) | 0.022 ± 0.047 (0.633) ^+^ | 0.111 ± 0.122 (0.361) |
|  | T4 | 0.029 ± 0.096 (0.765) | 0.001± 0.047 (0.991) ^+^ | 0.217 ± 0.122 (0.079) |
| **Log-H_2_O_2_** **[µM]** | T1 | - | - | - |
|  | T2 | 0.111 ± 0.304 (0.715) | 0.051 ±0.089 (0.570) | 0.157 ± 0.320 (0.624) |
|  | T3 | -0.343 ± 0.304 (0.258) | 0.132 ± 0.09 (0.142) | 0.211 ± 0.320 (0.510) |
|  | T4 | -0.153 ± 0.304 (0.615) | 0.218 ± 0.09 (0.014)* | 0.647 ± 0.320 (0.043)* |
| **Log-MDA** **[nM]** | T1 | - | - | - |
|  | T2 | -0.214 ± 0.339 (0.529) | -0.041 ± 0.126 (0.744) | 0.023 ± 0.161 (0.89) |
|  | T3 | -0.008 ± 0.337 (0.981) | -0.004 ±0.125 (0.972) | 0.078 ± 0.161 (0.63) |
|  | T4 | -0.489 ± 0.337 (0.147) | -0.057 ± 0.125 (0.650) | 0.049 ± 0.162 (0.76) |
| **Log-8-OHdG** **[μg/l]** | T1 | - | - | - |
|  | T2 | - | 0.044 ± 0.069 (0.517) | 0.109 ± 0.176 (0.536) |
|  | T3 | - | 0.057 ± 0.069 (0.410) | 0.171 ± 0.176 (0.332) |
|  | T4 | - | 0.135 ± 0.069 (0.049)* | 0.374 ± 0.176 (0.033)* |
