# Supplementary material for: Increase in oxidative stress levels following welding fume inhalation: a controlled human exposure study
Source: Part Fibre Toxicol. 2016 Jun 10;13:31. doi: 10.1186/s12989-016-0143-7 (PMC4901438; doi:10.1186/s12989-016-0143-7)

Additional file 5: Figure S1. Schematic of exposure day sample collection and questionnaire schedule. Control day follows same schedule, but replaces welding fume exposure with HEPA-filtered air exposure.


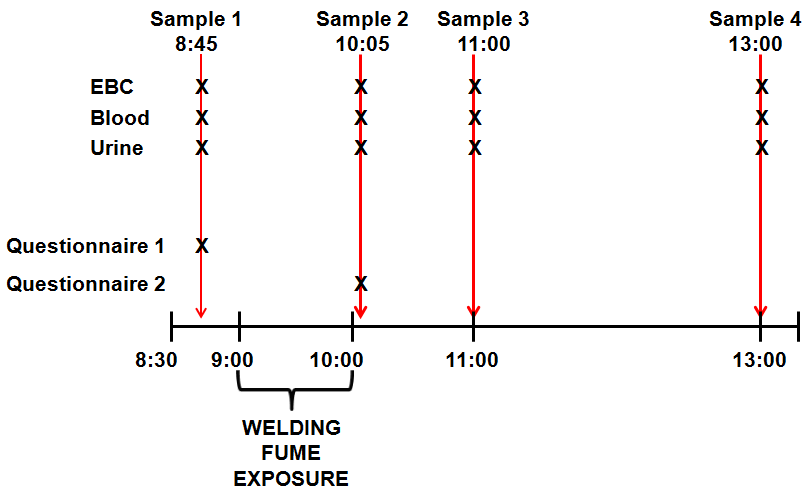

Supplement: Supplementary file 5 — Schematic of exposure day sample collection and questionnaire schedule. Control day follows same schedule, but replaces welding fume exposure with HEPA-filtered air exposure. (DOC 41 kb) [file 12989_2016_143_MOESM5_ESM.doc]
